# Clinical expert consensus document on rotational atherectomy from the Japanese association of cardiovascular intervention and therapeutics: update 2026

**DOI:** 10.1007/s12928-025-01233-3

**Published:** 2026-01-04

**Authors:** Kenichi Sakakura, Yoshiaki Ito, Yoshisato Shibata, Atsunori Okamura, Yoshifumi Kashima, Shigeru Nakamura, Yuji Hamazaki, Junya Ako, Hiroyoshi Yokoi, Yoshio Kobayashi, Yuji Ikari, Ken Kozuma

**Affiliations:** 1 Division of Cardiovascular Medicine, Saitama Medical Center, Jichi Medical University, 1-847 Amanuma, Omiya, Saitama, 330-8503 Japan; 2 Department of Cardiology, Saiseikai Yokohama City Eastern Hospital, Yokohama, Japan; 3 Department of Cardiology, Miyazaki Medical Association Hospital, Miyazaki, Japan; 4 Cardiovascular Center, Sakurabashi Watanabe Advanced Healthcare Hospital, Osaka, Japan; 5 Department of Cardiology, Oita Cardiovascular Hospital, Oita, Japan; 6 Sapporo Heart Center, Sapporo, Japan; 7 Cardiovascular center, Kyoto Katsura Hospital, Kyoto, Japan; 8 Division of Cardiology, Ootakanomori Hospital, Kashiwa, Japan; 9 Department of Cardiovascular Medicine, Kitasato University School of Medicine, Sagamihara, Japan; 10 Department of Cardiology, Fukuoka Sanno Hospital, Fukuoka, Japan; 11 Department of Cardiovascular Medicine, Chiba University Graduate School of Medicine, Chiba, Japan; 12 Department of Cardiology, Tokai University School of Medicine, Isehara, Japan; 13 Department of Cardiology, Teikyo University School of Medicine, Tokyo, Japan

**Keywords:** Rotational atherectomy, Rotablator, Complications, Slow flow, Burr entrapment, Perforation, Calcification

## Abstract

The Task Force on Rotational Atherectomy of the Japanese Association of Cardiovascular Intervention and Therapeutics (CVIT) proposed the expert consensus document to summarize the techniques and evidence regarding rotational atherectomy (RA) in 2020, which was updated in 2023. Because the revascularization strategy to severely calcified lesions has been the hottest topic in contemporary percutaneous coronary intervention (PCI), many literatures related to RA have been published since 2023. Latest advancements have been incorporated in this updated expert consensus document.

## Introduction

Severe calcification in atherosclerotic plaques has been the most common cause of poor clinical outcomes since the beginning of percutaneous coronary intervention (PCI) [1–4]. Rotational atherectomy (RA) has been widely used for severely calcified coronary lesions for more than 20 years to improve clinical outcomes in patients with severely calcified lesions. North American expert review as well as European expert consensus on RA was published to provide a clinical standard for RA operators [5, 6]. As compared to North America and European countries, RA in Japan has uniquely developed with the aid of greater usage of intravascular imaging devices [7–12]. Since the cost of intravascular ultrasound (IVUS) or optical coherence tomography (OCT) during percutaneous coronary intervention (PCI) has been covered by the government insurance system in Japan, RA operators could easily access to IVUS or OCT. IVUS has been used to understand the guidewire bias and to decide appropriate burr sizes during RA [13], whereas OCT can be used to measure the thickness of calcification during RA [14], which could result in appropriate burr size up [15]. Furthermore, the prevalence of PCI with RA has been higher in Japan than in other countries. In fact, the prevalence of PCI with RA was approximately 3.3% in Japan [16], which was similar to those in United Kingdom (3.1%) or France (2.9%) and was higher than those in Italy (1.3%) or Germany (0.8%) [6].

On the other hand, RA had not been allowed to any operators without on-site surgical back-up in Japan until April 2020. However, the government and the Japanese Association of Cardiovascular Intervention and Therapeutics (CVIT) worked together and released the new facility criteria for RA in April 2020, which allowed operators to perform RA without on-site surgical back-up. The new facility criteria have rapidly increased the number of RA operators in Japan. In fact, more than 300 facilities have completed the RA training program that was organized by CVIT and manufacturer (Boston Scientific Japan). Early experience from a newcomer facility was reported [17], suggesting the safety and feasibility of RA in newcomer facilities. The Task Force on Rotational Atherectomy of CVIT proposed the expert consensus document on RA to summarize the techniques and evidence regarding RA in 2020 and updated in 2023 [18, 19]. Because the revascularization strategy to severely calcified lesions has been the hottest topic in contemporary PCI, many literatures related to RA have been published since 2023. Latest advancements have been incorporated in this updated expert consensus document.

## Aim of RA

Before the stent era, the main purpose of RA was to debulk atherosclerotic plaques including calcification in coronary arteries [20]. However, the incidence of restenosis was considerably high following debulking using RA [21]. The emergence of drug-eluting stent (DES) has dramatically changed the indications of PCI, which includes diffuse long calcified lesions. The lesion modification, which facilitate the delivery and expansion of DES, would be the most frequent purpose in the contemporary RA, and the long-term outcomes of DES following RA was acceptable [22–28], except specific characteristics such as calcified nodule or hemodialysis [29–32]. Recently, 5-year outcomes of the PREPARE-CALC trial, which randomly assigned 200 patients 1:1 to modified balloons or RA before DES implantation, have shown the significant reduction of target lesion revascularization after RA [33]. The DIRO trial, which was a prospective randomized trial to compare stent expansion following RA versus orbital atherectomy (OA) under the OCT guidance, revealed that RA could produce a larger stent expansion than OA in heavily calcified lesions [34]. The ROTA.Shock trial, which was a randomized trial to compare plaque modification of severely calcified lesions by IVL with that of RA under the OCT guidance, showed that RA was associated with a greater acute lumen gain than IVL [35]. Furthermore, the ROLLER COASTR-EPIC22 trial, which was a randomized trial to compare the stent expansion among RA, intravascular lithotripsy (IVL), and excimer laser coronary angioplasty (ELCA), showed that IVL was noninferior to RA in terms of stent expansion, whereas ELCA did not reach this noninferiority margin compared with RA [36]. Recent meta-analysis showed that the efficacy was comparable between IVL and RA, but the incidence of complication was less in IVL than in RA [37, 38]. However, since IVL is a cracking device and RA is a debulking device, the role for severely calcified lesions would be different between IVL and RA.

The mid-term or long-term clinical outcomes following RA might not be satisfactory in specific characteristics such as hemodialysis or malnutrition [39–41], partly because the natural prognosis of patients with such characteristics would be worse than that without such characteristics. Incomplete stent apposition (ISA) is frequently observed in severely calcified lesions or calcified nodules even after the lesion modification by RA [42, 43], while the clinical significance of ISA in severely calcified lesions remains undetermined.

Moreover, RA might prevent the polymer damage, when DES was delivered to the calcified lesions [44]. Although the initial results of debulking using RA was not satisfactory, the debulking might have developed with the aid of imaging devices and drug-coated balloon in Japan [45–52]. Furthermore, the long-term outcomes following RA with plain old balloon angiography was acceptable when the target lesions were limited to large coronary arteries (diameter ≥ 3 mm) [53]. The combination of RA and modified balloons including cutting balloons is commonly used in clinical practice. However, clinical efficacy is comparable between the combination of RA and modified balloons and the combination of RA and plain balloon [54–56]. The combination of RA and OA may be applicable to modify the guidewire bias or to facilitate the safer use of large burrs [57–62]. Although the combination of RA and IVL has not been systematically examined [63], RA may facilitate the IVL catheter to reach the severely calcified lesions [64–67]. The Dual-Prep registry, which was a multicenter, prospective registry of consecutive image-guided PCI, applied RA prior to IVL in 83.9% cases and demonstrated that image-guided atherectomy followed by IVL lesion preparation was associated with high procedural success rates and satisfactory non-eccentric stent expansion [68]. CVIT also updated the consensus document regarding device indication for calcified coronary lesions, which allowed us to use the combination of atherectomy devices (RA or OA) and IVL [69].

The contemporary indications for RA are summarized in Table 1. Calcifications in computed tomography (CT) may predict the need for RA [70, 71]. In IVUS, calcifications are typically described as high echoic signals with an acoustic shadow behind the signal [72]. Reverberations that are concentric and arctic lines at equal intervals would be observed within the acoustic shadow [72, 73]. Because reverberations are closely associated with smooth surface of the calcified lesions [74], reverberations may be observed in the calcified lesions after RA as well as the unmodified calcified lesions with smooth surface. Recently, Jinnouchi, et al. reported the association between slow flow following RA and reverberations [75] and also reported that the high number of reverberation was significantly associated with stent underexpansion and ischemia-driven target lesion revascularization in PCI with RA [76]. However, the clinical significance of the natural reverberations has not sufficiently been investigated. A systematic review and meta-analysis compared the clinical outcomes between planned RA and bailout RA, and reported that planned RA resulted in significantly shorter procedural times, less contrast use, lesser dissection rates and fewer stents used as compared to bailout RA [77]. On the other hand, recently, Bacmeister, et al. analyzed more than 1000 lesions and reported that unplanned RA was associated with favorable outcomes when compared to planned RA [78]. Kanda, et al. reported that secondary RA strategy (balloon dilatation before RA) was inversely associated with the occurrence of prolonged ST-segment elevation in the comparison of primary RA strategy [79]. The CRATER trial is a prospective, multi-center, randomized study, and compared the costs, safety, and efficacy of elective-RA versus bailout-RA in severely calcified lesions in patients with chronic kidney disease [80]. In this randomized trial, although total healthcare costs were comparable between bailout-RA and elective-RA, a substantial number of patients required crossover from bailout-RA, highlighting the potential benefit of elective-RA [80].

**Table 1:** Contemporary indications for rotational atherectomy

Indications	Severely calcified lesions (typically 360 degree calcification)
	Napkin ring calcification
	Calcification showing reverberation in IVUS
	Device uncrossable lesions
	IVUS/OCT could not cross (relatively common)
	Microcatheter could not cross (relatively rare)
**Possible indications**	Moderately calcified lesions (> 180 degree calcification)
**High risk lesions**	Lesions with thrombus
	Lesions with extensive dissection
	Lesions with an angle
	Bypass graft lesions
**Contraindication**	Last remaining vessel with compromised left ventricular function

Abbreviations: IVUS = intravascular ultrasound, OCT = optical coherent tomography

## Junior RA operator

In this document, we defined junior RA operator as RA operator with less than 50 RA experiences. Because the number of RA cases per operator as well as the number of RA cases per year in the facility was inversely associated with adverse events [16, 81, 82], the number of RA cases per operator would be important to prevent severe complications. Moreover, the good indication for RA does not necessarily mean low-risk RA. For example, the ostial right coronary artery (RCA) lesions have been recognized a good indication for RA [83], whereas RA to the ostial RCA is known to be technically difficult [84]. Therefore, the recommendation for junior RA operators may be different from that for senior operators in some sections.

## Patient’s general conditions, mechanical support, temporary pacing

When we plan to perform RA, we should evaluate patient’s general conditions such as vital signs and cardiac functions. Since RA is often performed in the situation of complex and high-risk intervention in indicated patients (CHIP) [85–87], we should also evaluate patients clinical background and atherosclerotic risk factors [88]. In general, RA is feasible to the culprit lesion of acute coronary syndrome as a bailout procedure [89–93]. However, it should be recognized that emergent RA is closely associated with serious adverse events [16]. Although there was no evidence regarding blood pressure during RA, it would be important to keep systolic blood pressure (SBP) ≥ 120 mmHg (at least ≥ 100 mmHg) for the prevention of complications such as slow flow. If slow flow occurred in patients with left ventricular dysfunction, there would be a greater risk of hemodynamic collapse. If a patient with left ventricular dysfunction shows low SBP, we may consider to use mechanical supports such as intra-aortic balloon pumping (IABP) before RA [94]. Chen, et al. reported that the major adverse cardiac event rate was higher in RA with bailout IABP than in RA with primary IABP [95], implying that the use of IABP should be implemented at the beginning of RA if a complex procedure is anticipated. Although it is very rare to insert veno-arterial extracorporeal membrane oxygenation (V-A ECMO) for elective PCI with RA, it is an option for very high-risk PCI with RA to take additional arterial and venous sheathes just in case of emergent V-A ECMO. Moreover, if such patient would undergo elective RA, it is better to evaluate the abdominal and thoracic aorta by computed tomography (CT) to check the contraindications for IABP such as aneurysm or shaggy aorta. Although the Impella (Abiomed, Danvers, MA, USA) is not allowed to use as a support device for patients who undergo elective high-risk PCI in Japan [96], the Impella is considered to be an option as a support device for elective PCI with RA in USA [97]. If patients with cardiogenic shock already received the Impella or V-A ECMO supports, RA to the severely calcified lesions can be a bailout option from cardiogenic shock. Furthermore, although beta-blockers are cornerstone for optimal medical therapy, some operators hesitate to use beta-blockers, because of the possible risk of slow flow [98]. Of course, bradycardia (heart rate < 60 bpm) could be a problem during RA, beta-blockers can be continued, because the risk of slow flow was comparable between with and without beta-blockers [99].

Arrhythmia such as bradycardia or atrioventricular block sometimes happens in RA, especially during the treatment of RCA. Temporary pacing is a reliable option to continue procedures during arrhythmia. However, senior RA operators may not use temporary pacing by several reasons: [1] short ablation time may not induce sustained arrhythmia, [2] cough resuscitation may be effective for arrhythmia during RA [100], and [3] there is a risk of ventricular perforation induced by temporary pacing catheter [101, 102]. Nevertheless, it would be a safe approach for junior RA operators to insert temporary pacing for specific lesions. Brady-arrhythmia can occur during RA to RCA, dominant left circumflex (LCX), left main trunk, and rarely left anterior descending artery (LAD) lesions. In this document, we recommend junior RA operators to consider temporary pacing for RA to RCA, dominant LCX, and left main trunk lesions. Although intravenous aminophylline may be useful to prevent brady arrhythmias during RA [103–105], this consensus document does not recommend the use of aminophylline yet because of limited clinical experiences and concerns for adverse drug reactions.

Kusumoto et al. reported a unique case of trans-coronary pacing via RotaWire [55]. In this case report, the cathode of an external pacemaker was attached to the distal external end of the guide wire using a crocodile clip, whereas the anode was attached to the needle which is inserted under the skin of the anaesthetized groin [106]. Iqbal, et al. investigated the feasibility of transcoronary pacing during RA [107]. Their success rate of transcoronary pacing was 91.7% (121/132 RA cases) [107]. The possibility of temporary guidewire pacing can be discussed as potential substitutes for temporary pacing catheters. However, since there are several limitations such as coronary spasm or twitching diaphragm in temporary guidewire pacing, we do not recommend temporary guidewire pacing in this document yet.

## Guide catheter for RA

Although RA is possible either trans-radial, trans-femoral, or trans-brachial [108–110], RA operators should recognize the maximum burr size for each guide catheter size. A 6-Fr guide catheter can accommodate ≤ 1.75-mm burr, whereas a 7-Fr guide catheter can accommodate ≤ 2.0-mm burr. When RA operators consider ≥ 2.15-mm burr, a 8-Fr guide catheter is necessary. However, if there is severe tortuosity in a guide catheter, operators may feel strong resistance during advancing the burr in the guide catheter, which results in the burr-size down. Moreover, if operators make side-holes in a guide catheter by themselves, such handmade side-holes may prevent the burr from advancing in the guide catheter. For junior RA operators, it is important to check coronary flow more frequently than senior RA operators to notice the occurrence of slow flow or perforation immediately, which would be easier in ≥ 7-Fr guide than in 6-Fr guide because of the large dimeter of the drive shaft sheath (4.3 Fr).

The choice of guide catheter curves varies even among senior RA operators. Although the appropriate back-up support is important for stable procedures, the strongest back-up support, which is sometimes required for PCI to chronic total occlusion, would not be necessary for RA. The coaxial positioning of the guide catheter would be of utmost importance for successful RA. However, strong back-up support can be a key to success in limited cases [111]. Furthermore, if the guide catheter is floated around the aortic cusp, the burr may cause aortic valve injury and subsequent severe aortic regurgitation [112, 113]. The engagement of guide catheter is often difficult after transcatheter aortic valve replacement but is necessary to use atherectomy devices [114, 115].

## Guidewire for RA

Two types of guidewires for RA have been commercially available: RotaWire floppy (Boston scientific, Marlborough, MA, USA) and RotaWire extra-support (Boston scientific, Marlborough, MA, USA). Both RotaWires have 0.014-inch/0.36-mm spring tip and 0.009-inch/0.23-mm guidewire shaft. However, the length of the tapered segment is considerably different between RotaWire floppy and RotaWire Extra-support. RotaWire floppy has long tapered shaft (13 cm of < 0.0077-inch/0.20-mm shaft) and short spring tip (22 mm), whereas RotaWire Extra-support has short tapered shaft (5 cm of < 0.009-inch/0.23-mm shaft) and long spring tip (28 mm) [116]. For successful RA, it is important not to make a bend in RotaWires, because a bend in RotaWires substantially increases the friction force between burr and RotaWire. Therefore, the use of microcatheter is recommended to bring RotaWires to the target. RA operators should use the conventional 0.014-inch guidewire to cross the lesion, and then exchange the conventional guidewire to the RotaWire by using microcatheter. Recently, the manufacturer (Boston scientific, Marlborough, MA, USA) refined both RotaWire floppy and RotaWire Extra-support, and introduced both RotaWire drive floppy and RotaWire drive extra-support [117]. The torque response has considerably improved in both RotaWire drive floppy and extra-support. However, we still recommend using the conventional 0.014-inch guidewire to cross the lesion, and then exchange the conventional guidewire to the RotaWire drive by using microcatheter not to make a bend in RotaWires. The guidance for selection of RotaWires is shown in Table 2.

**Table 2:** Guidance for selection of RotaWires

Characteristics or specific situations	RotaWire Floppy or Extra-Support
Ability to ablate the severely calcified plaques (Ablation efficiency)	Extra-Support > Floppy
Ability to straighten the tortuous coronary artery	Extra-Support > Floppy
Ability to strengthen the back-up force in the system	Extra-Support > Floppy
When pre-intravascular imaging devices cross the lesion and provide sufficient information regarding the guidewire bias	Select either Extra-Support or Floppy according to the information from imaging devices and angiography
When operators cannot judge the guidewire bias from angiography and/or intravascular imaging	Floppy first
When junior RA operators cannot understand which RotaWires are more suitable to the lesion	Floppy first
When the burr cannot cross the lesion, the exchange from Floppy to Extra-Support	May work well, because of the change of the contact point. However, the strong guidewire bias may cause deep ablation.
When the burr cannot cross the lesion, the exchange from Extra-Support to Floppy	May work well, because of the change of the contact point.

## Appropriate burr size

In early experiences with RA, big burrs were used to debulk the calcified plaques. However, a randomized trial comparing small burrs (burr/artery ratio of ≤ 0.7) with large burrs (burr/artery ratio of > 0.7) revealed that small burrs achieved similar immediate lumen enlargement and late target vessel revascularization compared with large burrs, but showed fewer complications [118]. Furthermore, a recent retrospective study reported that bur/artery ratio > 0.61 was associated with worse clinical outcomes [119]. European expert consensus document recommend burr/artery ratio of 0.6 [6], and North America expert consensus document recommend burr/artery ratio of 0.4–0.6 [5]. In this document, we recommend burr/artery ratio of 0.4–0.6 without intravascular imaging devices, and recommend using intravascular imaging if RA operators aim to achieve burr/artery ratio of ≥ 0.6.

For lesion modification, single burr may be sufficient to facilitate stent delivery and stent expansion. However, second burr is sometimes necessary even for lesion modification. The guidance for second burr is summarized in Table 3.

**Table 3:** Guidance for use of the second burr

Size down or size up	Situations	Comments
**Size down**	When the first burr cannot cross the lesion, operators should size down with the second burr.	This size down is important to prevent severe complications. It is better for junior RA operators to consider size down, when the burr could not cross the lesion after 4–6 RA sessions. If there are signs of slow flow such as chest pain or ECG changes, immediate size down should be considered.
**Size up**	When operators aim to use the big burr (≥ 1.75-mm), start with the small burr (≤ 1.5-mm) for safety, and then size up to the big burr.	Generally, the range of size up would be 0.25 mm to 0.75 mm. Junior RA operators should select the 0.25-mm or 0.50-mm size up.
**Size up**	Operators started with the first burr, and checked intravascular imaging after the pass of the first burr. Intravascular imaging revealed the insufficient ablation, which recommended the size up.	The thickness of calcification derived from OCT may be helpful to decide the necessity of size up. Before size up, operators should check signs of slow flow such as chest pain or ECG changes.
**Size up**	Operators finished RA with the first burr, and then balloon (non-compliant balloon, scoring balloon, or cutting balloon) dilatation was tried. However, the lesion was not dilated sufficiently (typically dog-bone phenomenon), and then size up to the big burr.	Careful manipulation of RotaWire is necessary, because there would be some dissections after balloon dilatation.
**Size up**	During RA, no additional speed down was observed in spite of the forceful manipulation of the burr. Operators judged that the burr did not contact to the calcification adequately, and then sized-up to increase the contact area.	No additional speed down in spite of forceful manipulation of the burr is the high-risk situation for burr entrapment or perforation.

## Burr manipulation and rotation speed

A pecking motion (quick push-forward/pull-back movement of the burr) has been a standard burr manipulation in RA [6]. Although several burr manipulations have been conducted by RA experts, the common part of burr manipulation is to push-forward from the platform and pull-back the burr to the platform. The speed of manipulation varies widely among experts. Some experts prefer very quick, whereas other experts prefer very slow. Either speed is acceptable as long as the following points are considered: [1] Operators should control the burr’s motion. If operators feel difficulty to control the burr’s motion, the speed may be too quick, [2] operators should avoid excessive rotational speed down, and [3] operators should not deactivate the system when the burr is in the middle of stenosis, which may result in the entrapment of the burr. Operators should deactivate the system when the burr was pulled-back to the platform. When the burr is parked at the platform, operators need to pay attention to ischemia caused by the burr at the platform. If the burr size is similar to the vessel diameter at the platform, ischemia easily occurs during the parking at the platform. Since recent fluoroscopy systems have the function of recording the fluoroscopic images as a movie, it is reasonable for junior RA operators to record the fluoroscopic movie of each RA session and to review the previous fluoroscopic movie just after each session. This allows operators to review the movement of the burr, RotaWire, and guide catheter. Also, operators may have a chance to recognize the early signs of burr disconnection [120, 121]. Furthermore, the ROTAPRO™ system (Boston scientific, Marlborough, MA, USA) has been gradually introduced to newcomer facilities as well as existing facilities in Japan. In brief, the ROTAPRO™ system is composed of a digital console and an advancer including buttons to activate devices [117]. The ROTAPRO™ system eliminated the foot pedal.

Duration of individual runs is also important to prevent complications. The manufacturer recommends the duration of individual runs less than 30 s. In general, longer duration would be associated with greater amount of debris. For junior RA operators, short duration (e.g. ≤l5-20 s) for a single session would be recommended. Furthermore, it is important to check the situations such as ECG and vital signs between the sessions.

Regarding the rotational speed, since the manufacturer set the minimum speed as 140,000 rotations per minute (rpm) and the maximum speed as 190,000 rpm [122], this consensus document also recommends to use 140,000 to 190,000 rpm, and may consider to use > 190,000 rpm when operators feel difficulty to cross the lesion. There was a debate whether low rotational speed can reduce the risk of slow flow. Platelet aggregation was greater in high-speed (180,000 rpm) than in low-speed (140,000 rpm) in an early experiment in vitro [123], which has not been proved in vivo. A randomized control study comparing low-speed (140000 rpm) with high-speed (190000 rpm) revealed that the incidence of slow flow was similar between low-speed and high-speed [124]. Therefore, it is not reasonable to use low-speed for the prevention of slow flow. On the other hand, there were several interesting findings from Japan regarding the additional lumen gain in low-speed RA. Mizutani, et al. reported that the greater debulking area following low-speed (< 150000 rpm) was confirmed by OCT [125]. Yamamoto, et al. also reported that the greater debulking area following very low speed (110000 rpm) was confirmed by OCT [126]. However, Kobayashi, et al. reported that there were no additional lumen gain following low speed (120000 rpm) [127]. Considering the above evidence, low speed (140000 rpm) within the instructions for use could be an option to acquire additional lumen gain, but very low speed (< 140000 rpm) should not be used, especially for junior RA operators.

The very-high speed (> 190000 rpm) is sometimes used in Japan [14, 128]. A bench test showed that the RotaWire may be spinning under the maximum rotational speed [129], while the RotaWire theoretically would not spin during high-speed mode because of the internal brake and WireClip. The spinning of RotaWire may be associated with the guidewire failure [130, 131], which has not been proved in the large registry data.

## How to bring the burr to the platform

Before activating the burr, it is a key to success to bring the burr into the platform with keeping the RotaWire stable position. However, several troubles were frequently observed in the above process. The RotaWire could advance too deep or be pulled-back. The RotaWire may make a loop at the outside of the guide catheter, which can result in severe complications [132]. Although the manufacturer recommends using the dynaglide mode only when operators remove the burr, not a few RA operators use the dynaglide mode when operators bring the burr to the platform. In this document, we would like to show the risk and benefit of both ways (using dynaglide or not) in Table 4. Because both ways have some disadvantages, operators need to understand both ways to avoid possible complications.

**Table 4:** Advantages and disadvantages of using dyna glide mode when operators bring the burr to the platform

	Using dynaglide mode	No dynaglide mode
Extent of resistance, when operators advance the burr	Less	Greater
RotaWire tends to advance more distally, when operators advance the burr	No	Yes
RotaWire tends to be pulled back, when operators advance the burr	Usually no, but possibly yes when the coaxiality of guide catheter was not maintained	Usually no, but possibly yes when assistant pulled the wire too much
Possibility of making a loop at the outside of the guide catheter	Very rare	Yes (very dangerous if operators could not notice it)
Combination between an operator and an assistant	Not important	Important (an assistant have to control the RotaWire during advancing the burr)
Jumping phenomenon, when operators activate the burr at the platform	Rare	Yes, therefore it is important to fix a nob at 1–2 cm apart from the end in advancer
Damage to the inner lumen of the guide catheter	Possible	No

## Rota cocktail

RA advancer has a saline infusion port. Although the instructions for use does not recommend using any drugs into the saline bag, various drugs have been used to prevent slow flow. A representative combination of drugs was verapamil 10 mg (5 mg), nitroglycerin 5 mg (2.5 mg), heparin 10,000 unit (5000 unit), and saline 1000 ml (500 ml). Another representative combination of drugs was nicorandil 24 mg (12 mg), nitroglycerin 5 mg (2.5 mg), heparin 10,000 unit (5000 unit), and saline 1000 ml (500 ml). Two randomized studies compared nicorandil based cocktail with verapamil based cocktail and showed that the incidence of slow flow was significantly lower in the nicorandil based cocktail than in the verapamil based cocktail [133, 134]. Furthermore, the cocktail including aminophylline may prevent bradycardia during RA to RCA or dominant LCX [135]. Recently, Guo, et al. conducted a randomized study to compare the incidence of slow flow between simple cocktail (only heparin and saline) and traditional cocktail (heparin, verapamil, nitroglycerin, and saline) [136]. The incidence of slow flow tended to be higher in the simple cocktail group without reaching statistical significance but the incidence of coronary spasm was significantly higher in the simple cocktail group than in the traditional cocktail group [136]. Moreover, You, et al. conducted a randomized study to compare myocardial injury after RA between low-temperature RA-flush solution and normal-temperature RA-flush solution and showed that some parameters of myocardial injury was less in low-temperature RA-flush solution than in normal-temperature RA-flush solution [137]. Preferred cocktail varied widely among RA experts, partly because intra-coronary injection of vasodilators such as nicorandil or nitroprusside was easily performed in the contemporary catheter laboratories. Either combination of drugs are acceptable, as long as intra-coronary injection of vasodilators is available in a catheter laboratory. If only saline is used for RA, the activated clotting time (ACT) should be checked before RA to prevent possible thrombus formation. Although the ideal ACT during RA is unknown in literatures, heparin should be administered to maintain the ACT > 300 s or at least 250 s during RA [138].

## Imaging devices in RA

Imaging devices such as IVUS or OCT are useful in RA [139–146]. In this document, we recommend using IVUS or OCT before, during, and after RA. Since both IVUS and OCT have advantages and disadvantages, each device should be selected according to the purpose of RA in each case. The advantages and disadvantages of IVUS and OCT are summarized in Table 5. Since OCT needs to eliminate red blood cells, it is difficult to use OCT safely when dissection or hematoma occurred following RA [147]. Kawaguchi, et al. reported the impact of the degree of guidewire bias in the vessel’s healthy portion on coronary perivascular trauma in RA [148], which suggests the greater risk of vessel perforation when the intravascular imaging catheter is pushing the normal vessel to distort the vessel structure (tenting phenomenon). RA to the point of tenting phenomenon results in vessel injury or perforation [149]. On the other hand, Matsumura, et al. reported that reverse tenting sign in IVUS was associated with vessel injury after RA [150]. Reverse tenting sign, which was defined as the IVUS image of the healthy vessel segment being drawn into the lumen, might be caused by minor accordion phenomenon [150]. Moreover, Hashimoto, et al. also reported that there is a higher risk of medial injury due to the RA procedure, especially near the bifurcation of the left anterior descending artery and diagonal branch when the guidewire and IVUS catheter are close to the healthy side of the vessel wall [151]. Recently, Hashimoto, et al. reported that the artificial intelligence-based algorithm can use information from pre-IVUS images to accurately predict regions debulked by RA [152]. Hamana, et al. showed that optical frequency domain imaging (OFDI)-based simulation of RA effect is feasible while accuracy may be affected by the OFDI catheter and wire position [153]. When the guidewire bias is not appropriate for RA, operators can try to change the guidewire bias by [1] change the guidewire (Floppy to Extra-Support or vice versa), [2] change the guide catheter position or type of guide catheters, or [3] change the route and the depth of guide wire (from left circumflex main branch to obtuse marginal branch or from posterior descending branch of RCA to posterior lateral branch of RCA). Despite the above efforts, the guidewire bias may be unfavorable for RA. In such situations, the switch from RA to intravascular lithotripsy or modified balloons is an alternative option. In limited cases, the aggressive wire recanalization in calcified atheroma and dilatation (ARCADIA) technique may be useful to treat calcified nodule [154, 155].

Although intravascular imaging before RA can provide useful information regarding appropriate burr size or RotaWire, intravascular imaging catheter may not cross the severely calcified lesions. If pre-RA imaging is critical for safe RA (e.g. ostium of left circumflex lesions), the use of guide-extension catheters or small balloon pre-dilatation may be considered to cross the lesion, while large balloon dilatation may make unfavorable dissection for RA [156]. For imaging device uncrossable lesions, small burrs (1.25-mm or 1.5-mm burrs) would be the choice. Either 1.25-mm or 1.5-mm burr is to be decided at operator’s discretion. Sakakura, et al. compared the complications between 1.25-mm and 1.5-mm burrs for IVUS-uncrossable lesions and showed the incidence of complications was comparable [157]. Moreover, the risk of complications was greater in imaging device uncrossable lesions than in imaging device crossable lesions [158]. Junior RA operators should be careful about those imaging device uncrossable lesions. Small balloon dilatation before RA can be an option for junior RA operators to prevent severe complications.

Maehara and colleagues developed the IVUS calcium score as well as the OCT calcium score [159, 160]. The OCT calcium score was composed of maximum calcium angle (> 180º = 2 points), maximum calcium thickness (> 0.5 mm = 1 point), and calcium length (> 5 mm = 1 point) [159]. The OCT calcium score was closely associated with stent underexpansion [159], which suggests that the lesions with high OCT calcium score would require RA to achieve optimal stent expansion. The IVUS calcium score was composed of length of superficial calcification > 270º (≥ 5 mm = 1 point), presence of 360º superficial calcification (yes = 1 point), presence of calcified nodule (yes = 1 point), and vessel diameter (< 3.5 mm = 1 point) [160]. They advocated that operators should consider the use of atherectomy devices to avoid stent underexpansion when the target lesion’s IVUS calcium score was ≥ 2 points [160]. The CORAL study reported that calcified lesions with OCT-calcium score of 1–2 were optimally treated with a balloon-only preparation strategy using a non-compliant/scoring/cutting balloon [161]. Recently, the OCT calcium score has been updated [162]. Although both IVUS and OCT calcium scores have a potential impact on the indication for RA, intravascular imaging catheters may not cross the severely calcified lesions before RA. These calcium scores may be more applicable to decide the indication for the burr size up or other aggressive lesion modifications including IVL after the small burr cross the lesion rather than to decide the indication for RA.

**Table 5:** Comparison of intravascular imaging in RA between IVUS and OCT

	IVUS	OCT
Strong points	• Since IVUS dos not need to eliminate red blood cells, operators can use IVUS safely when dissection or hematoma occurred following RA.	• OCT can provide the more detailed information regarding calcification such as thickness of calcification.
Weak points	• IVUS cannot prove the thickness of calcification.	• Difficult to use for ostial RCA or ostial LMT lesions. • Since OCT need to eliminate red blood cells, it is difficult to use OCT safely when dissection or hematoma occurred following RA. • Need to be careful for volume overload following multiple observations, especially for patients with low cardiac function. • May miss calcification when soft tissue buried the calcification
Judgement of guidewire bias	• Accurate, but IVUS probe tends to separate from the guidewire when operators push an IVUS catheter. Before checking the guidewire bias, operators should pull the IVUS catheter a bit to correct the separation between the guidewire and IVUS probe.	• Accurate
Efficacy versus Safety	• IVUS would increase the safety in RA. IVUS can be used in severe complications following RA, and the role of IVUS would be critical in some situations.	• OCT would increase the efficacy such as aggressive ablation in RA.

## Endpoint of RA

Operators should set an appropriate endpoint of RA for each case. In general, when a burr crosses a lesion without any resistance and no additional speed down is observed, operators can finish RA unless operators consider burr size-up (conventional/classical endpoint of RA). In the contemporary PCI, the endpoint of RA may become more complex, owing to the development of IVUS/OCT. If operators use IVUS before RA, the crack in the napkin-ring calcification can be an endpoint of RA [13]. If operators use OCT before RA, the residual thickness of calcification or dissection can be an endpoint of RA [14, 163]. However, operators, especially junior RA operators, should not stick to the conventional/classical endpoint of RA, if there are signs of slow flow or other complications.

## Complications: slow flow

Slow flow is the most frequently observed complication following RA. The incidence of slow flow was approximately 5 to 20% [124, 134, 164], and varied widely among literatures, partly because the timing of judgement (just after RA or final shot) and the definition of slow flow were different among literatures. Lesion length and burr-to-artery ratio were reported as the determinants of slow flow [124, 165]. Furthermore, IVUS findings such as longer lesion length, the maximum number of reverberations, and the greater arc of calcification at MLA may predict slow flow after RA [75]. For the prevention of slow flow, appropriate burr size, short ablation time, and gentle manipulation avoiding excessive speed down would be important to minimize the amount of debris caused by RA. A retrospective study revealed that short single session (≤ 15 s) was inversely associated with slow flow after RA [165], which suggests that short single session can reduce the incidence of slow flow after RA. However, a randomized control study to compare the incidence of slow flow following RA between the short single session strategy and the long single session strategy did not show the benefit of short single session [166, 167]. In brief, 266 patients were randomly assigned to the Short single session group (*n* = 132) or the Long single session group (*n* = 134) [167]. The incidence of slow flow just after RA was similar between the 2 groups (Short single session:14.4% versus Long single session: 14.9%, *p* > 0.999) [167]. The possible reasons for the discrepancy between the retrospective study and the randomized study should be noted. Since there is a time gap in the patient enrollment period between the retrospective study (November 2014 to August 2020) and the randomized study (April 2022 to February 2025), the operators engaged in the randomized study might increase the understanding of slow flow, follow the recommended techniques described in the clinical expert consensus document on RA from CVIT, and develop their skills in the prevention of slow flow [168, 169]. Furthermore, because there was no restriction regarding the time interval between sessions, operators could take sufficient time between sessions when ST- segment elevation was observed during RA. Because ST-segment elevation often precedes slow flow in RA, slow flow might have been prevented by the appropriate actions for ST-segment elevation such as sufficient interval, maintaining blood pressure, intracoronary vasodilators, burr size down, and intentional halftime [169].

Although there were no literature, some experts prefer to flash saline for the prevention of slow flow during RA. Moreover, it is important to keep sufficient systolic blood pressure ≥ 120 mmHg (at least 100 mmHg). If patient’s cardiac function is normal, sufficient hydration is also important. If a patient shows low blood pressure under poor cardiac function, IABP may be considered. Noradrenaline diluted in saline is frequently used to keep blood pressure. If operators took a venous sheath from femoral vein as a rescue sheath, such sheath would be helpful to inject the drug immediately. Unlike slow flow during primary PCI, slow flow during RA is gradually developed (i.e. TIMI-3 flow to TIMI-2 flow, then TIMI-2 flow to TIMI-1 flow, etc.) as long as operators do not ablate lipid-rich plaques. Therefore, it is important to watch ST-elevations in ECG, which usually antecedent slow flow. Appropriate actions for ST-elevations should be taken even if the patients are asymptomatic. If the TIMI-2 slow flow occurs, RA should be stopped temporarily until the TIMI-3 flow was restored. Most transient TIMI-2 slow flow would not result in periprocedural myocardial infarction if treated immediately [170]. However, if ST-elevation or chest pain after slow flow is prolonged, it would be important to halt RA and take a sufficient time until recovery. When ST-elevation or chest pain persists despite taking a sufficient time for recovery, it is an option to finish PCI as suboptimal revascularization and reschedule the staged-PCI if the risk of acute coronary closure is deemed to be low, because myocardial injury after slow flow is associated with long-term worse outcomes [171]. If blood pressure falls following slow flow, noradrenaline diluted in saline is injected to restore blood pressure. If noradrenaline did not work, the prompt insertion of IABP would be the next option. Intra-coronary vasodilators such as nitroprusside, nicorandil, and nitroglycerine are used to treat slow flow. Although there were no literatures comparing the efficacy among such vasodilators, nitroprusside may be the most potent vasodilator for slow flow [172]. Although nicorandil may be more effective than nitroglycerine [173], the rapid injection of nicorandil may provoke fatal arrhythmia or even cardiac arrest [174]. Moreover, the effect of intracoronary vasodilators would be weakened when the burr is parked within coronary artery or guide catheter, because the presence of burr, drive shaft sheath, and side hole in a guide catheter would prevent vasodilators from reaching to the vascular bed of coronary arteries. The use of microcatheters or double lumen catheters would be considered to minimize the risk of vasodilator-induced hypotension. Appropriate timing of intra-coronary vasodilators would be important to treat slow flow. Operators should check ECG or vital signs between sessions and check the flow when the change of ECG is observed. In case of severe slow flow (TIMI-0), the use of thrombectomy catheter can be considered before the injection of intra-coronary vasodilators. Thrombus formation might be one of causes of slow flow following RA [175]. The prevention and bailout for slow flow are summarized in Table 6. Recently, several groups reported that coronary microcirculatory resistance after RA was associated with long-term clinical outcomes [176–178]. However, the association between slow flow and coronary microcirculatory resistance after RA remains unconclusive.

**Table 6:** Prevention and bailout for slow flow

Prevention or bailout	Concept	Specific methods
Prevention	Do not make a large amount of debris	• Appropriate burr size • Avoid excessive speed down
Prevention	Maintain sufficient blood pressure	• Keep SBP ≥ 120 mmHg (at least 100 mmHg) • Use of diluted noradrenaline • Consider IABP when low SBP is derived from low cardiac function.
Bailout	Immediate treatment is most important	• Check the change of ST-segment, vital signs, and symptom (chest pain) between sessions. • Use of diluted noradrenaline if SBP fall • Use of intra-coronary vasodilators such as nitroprusside

Abbreviations: SBP = systolic blood pressure, IABP = intra-aortic balloon pumping

## Complications: Perforation/Rupture

Coronary perforation due to the burr is the most serious complication in RA, and the incidence of perforation in RA is approximately 1% to 2% [179–182]. If coronary perforation occurs during RA, there would be a considerable risk of in-hospital death, especially the lesions involving left main coronary artery [183]. The risk of perforation highly depends on the lesion characteristics such as vessel tortuosity or eccentricity of calcification. Since the shape of each burr is ellipsoid [184], the RA burr cannot follow the sharply angulated vessel, which results in the greater risk of perforation. The risk of perforation is generally considered to be greater in an eccentric calcification such as calcified nodules than in a concentric calcification such as napkin-ring calcification. Thus, operators need to be careful about RA to an eccentric calcification. The selection of appropriate burr size and RotaWire should be important to prevent perforation, and the use of intravascular imaging devices would help operators to select appropriate burr size and RotaWire. If intravascular imaging shows the finding that the guide wire is pushing the normal vessel to distort the vessel structure, the risk of vessel perforation/injury would be greater following RA [148]. If intravascular imaging devices could not cross the lesion, small burrs (1.25-mm or 1.5-mm) should be the choice, especially for junior RA operators. In general, a RotaWire floppy would follow the vessel without distorting the vessel configuration, whereas a RotaWire Extra-support would follow the vessel with distorting the vessel configuration. Therefore, the route of the burr would be considerably different between RotaWire floppy and RotaWire Extra-support, which suggests the importance of the choice of RotaWires for the prevention of perforation. If the guidewire bias was difficult to anticipate by intravascular imaging or angiography, a RotaWire floppy would be the choice.

The bailout of perforation caused by RA is basically similar to that caused by PCI without RA except the fact that operators have to remove the RA system with keeping the RotaWire. If operators lost the RotaWire following massive perforation, there would be no guarantee of recrossing the guidewire. A Kusabi trapping balloon (Kaneka, Osaka, Japan) can be used for the retrieval of ≤ 1.5-mm burrs in ≥ 7-Fr systems [185]. A balloon catheter or a perfusion balloon catheter should be promptly delivered to the lesion to seal the blood flow toward pericardial space. Since the pericardiocentesis is probably necessary in perforation following RA, the preparation for pericardiocentesis is important in catheter laboratories using RA. Multiple covered stents may be necessary to seal the blood flow completely [186–188]. The use of guide extension catheter or the use of double guide catheters may be considered to facilitate the delivery of covered stents [189, 190], because there would be a risk of dislodgement of covered stents in severely calcified lesions. In the use of double guide catheters, a balloon via the first guide catheter can be used to seal bleeding during the preparation of covered stent via the second guide catheter, and the same balloon can be used as the distal anchor balloon to facilitate the covered stent delivery. Moreover, operators should contact cardiovascular surgeons immediately just in case of unsuccessful percutaneous bailout. Since delayed cardiac tamponade and late-onset coronary artery aneurysm can occur after coronary rupture following RA [191], careful follow-up is warranted for patients who had coronary rupture following RA. Because the elevation of right atrial pressure is a more sensitive sign for cardiac tamponade than the change of systemic blood pressure or heart rate, continuous monitoring of right atrial pressure in ICU setting may be considered when there is a substantial risk of late tamponade [191]. The prevention and bailout for coronary perforation are summarized in Table 7.

**Table 7:** Prevention and bailout for coronary perforation/rupture

Prevention or bailout	Concept	Specific methods/comments
Prevention	Risk assessment is of utmost importance for prevention of perforation following RA.	• Greater risk in lesions with an angulation. • Risk of perforation is greater in eccentric calcification than in concentric calcification.
Prevention	Use appropriate burr size, and select appropriate RotaWires	• Do not push the burr too much, just deliver the bur to the lesion. • Small burrs (≤ 1.5-mm) would be the choice for the high risk lesions. • Interpretation of guidewire bias derived from intravascular imaging would be important to select appropriate RotaWires
Bailout	Keep the RotaWire within the lesion, when perforation occur.	• Do not be panic. Remove the Rota system with keeping the RotaWire within the lesion. • Covered stents and pericardiocentesis would be necessary in most cases. • Contact cardiovascular surgeons immediately in case of unsuccessful percutaneous bailout.

## Complications: burr entrapment

Burr entrapment is a unique complication in RA, and the incidence of burr entrapment is not derived from multicenter registries, but is available from single center studies ranging 0.4% to 0.8% [192–194]. Burr entrapment can occur from several mechanisms. One is called as “Kokesi phenomenon” that the burr was trapped in the distal portion of the proximal narrowing [193]. The mechanism of Kokesi phenomenon is considered to be that a friction heat enlarges the orifice and the coefficiency of friction in motion is smaller than that of friction at rest [193]. This type of burr entrapment may occur following forceful manipulation with small burrs. Another mechanism is the burr entrapment related to the vessel angulation. Since the shape of the burr is ellipsoid and the diamond coating is not available at the tail of the burr, the burr can be trapped by non-massive calcification at the site of angulation. In order to prevent burr entrapment, operators need to be careful about rotational speed reduction, sound of ablation, and resistance during the burr manipulation [195]. In the beginning of the burr entrapment, operators cannot pull the burr but sometimes can advance the burr. If operators can advance the burr safely beyond the point of the entrapment, there is a chance to pull back the burr using Dynaglide mode, which may result in successful bailout [196].

**Fig. 1:**
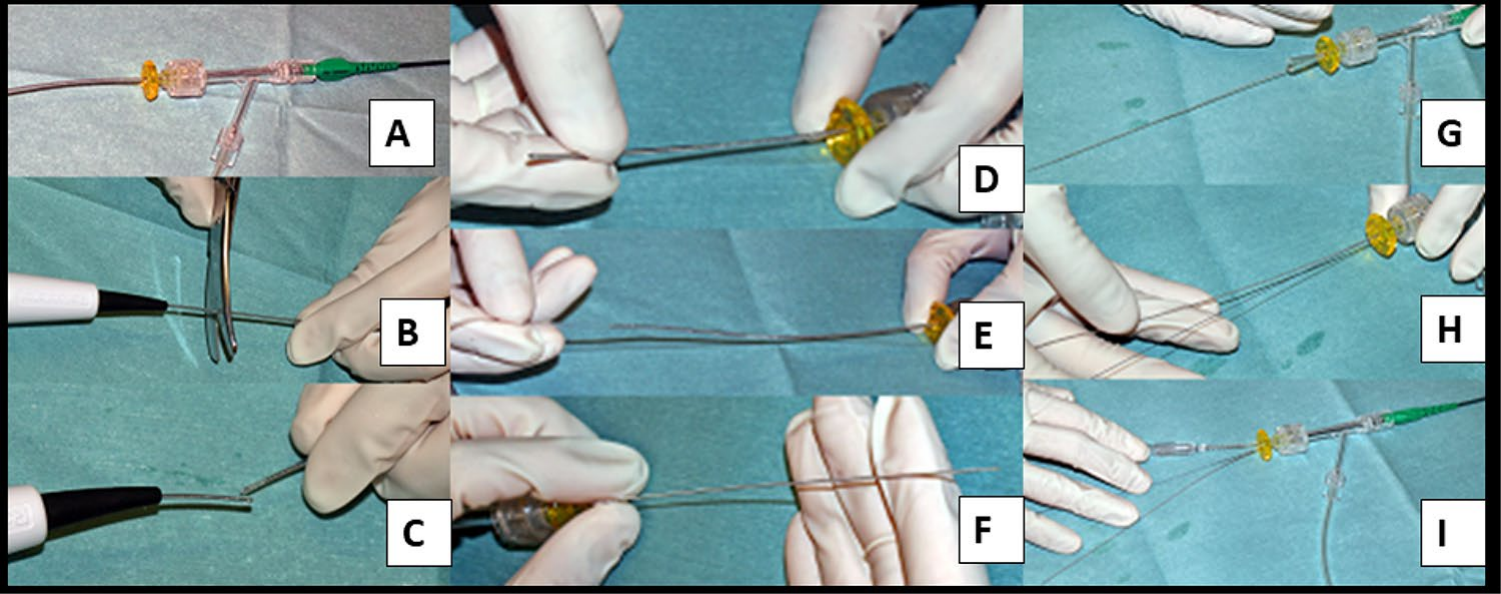
. How to cut and pull the drive shaft sheath. Panel A: A RA burr (1.25-mm) was inserted into a 6-Fr guide catheter via a Y connector. Panel **B**,** C**: The drive shaft, drive shaft sheath, and RA wire were cut together near the advancer. Panel **D**,** E**: The drive shaft sheath was pulled back and removed. Panel **F**: After the drive shaft sheath was removed, the drive shaft remained in the same position. Panel **G**,** H**: A guide wire (0.014 inch) passed through the guide catheter via an inserter and Y-connector. Panel **I**: A 2.5 × 15 mm conventional balloon easily passed through the guide catheter. This figure was reproduced with the permission from Sakakura, et al. [198]

**Fig. 2:**
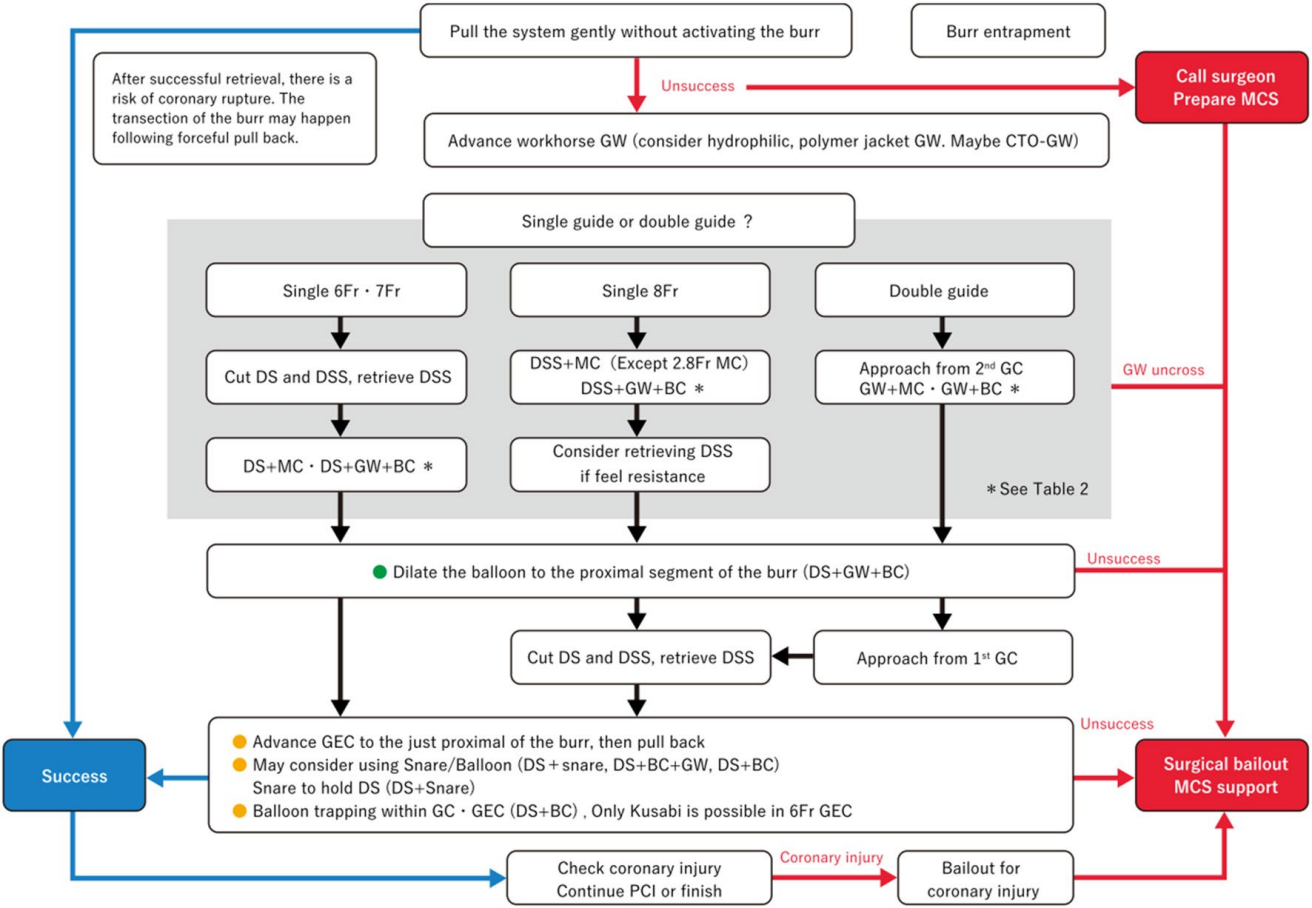
Flow chart for burr entrapment. Abbreviations: DS, drive shaft; DSS, drive shaft sheath; GW, guidewire. This figure was reproduced with the permission from Ogawa, et al. [207]

When the burr is entrapped, simple pull back may work as an initial bailout strategy, especially in the case of mild entrapment. However, since pull back at the point of entrapment may cause the transection of the burr [197], operators should not stick to simple pull back. If operators encounter the burr entrapment, it is important to assess the situation calmly. The presence of antegrade flow, ST-segment elevations in ECG, and patient’s chest pain should be evaluated. If there is no antegrade flow beyond the entrapped burr, the percutaneous bailout would be very difficult and be limited to experienced senior RA operators. In the meantime, operators should contact cardiovascular surgeons to discuss the surgical bailout. If the antegrade flow is present without ST-segment elevations, operators would have a time to consider the percutaneous bailout techniques. Although there have been several percutaneous bailout techniques in literatures [198–205], the main difference among various techniques was whether to use additional guide catheter (double guide catheters) or not (single guide catheter). If operators selected the double guide systems, operators would insert the conventional guidewire from the second guide catheter, and then would try to dilate the proximal part of the entrapped burr using a balloon [199]. If operators selected the single guide system, the next step would depend on the guide catheter size (≥ 8 Fr or ≤ 7 Fr). If operators used a ≥ 8Fr guide system, operators would insert the conventional guidewire, and then would try to dilate the proximal part using a balloon. However, if operators used a ≤ 7 Fr guide system, operators need to cut and pull the drive shaft sheath (Fig. 1) [198], because a ≤ 7 Fr guide catheter cannot accommodate the drive shaft sheath, guidewire, and balloon catheter together. After pulled out the drive shaft sheath, operators can try to dilate the proximal part using a balloon. Once operators pulled out the drive shaft sheath, operators can use inner catheters such as guide extension catheters [200, 203, 204]. Of course, operators should recognize the possibility of unsuccessful percutaneous bailout, and need to prepare the massive perforation or severe dissection following the burr retrieval [206]. The prevention and bailout for burr entrapment are summarized in Table 8 and are illustrated as Fig. 2 [207].

**Table 8:** Prevention and bailout for burr entrapment

Prevention or bailout	Concept	Specific methods/comments
Prevention	Risk assessment is of utmost importance for prevention of perforation following RA.	• Do not push the burr too much, just deliver the bur to the lesion. • Greater risk in lesions with an angulation. • Be careful about rotational speed deceleration, sound of ablation, and resistance during the burr manipulation.
Prevention	Do not inactivate the burr in the middle of the calcified stenosis	• There is no diamond coating at the tail of the burr • Moderate stenosis at the proximal of the target can be a cause of burr entrapment
Bailout	It is important to assess the situation such as the presence of antegrade flow, calmly.	• Do not activate the burr after burr entrapment. • Bailout techniques are divided to single guide bailout or double guide bailout. • Contact cardiovascular surgeons immediately in case of unsuccessful percutaneous bailout.

## Complications: transection of the RotaWire

The transection of the RotaWire is a rare complication in RA, and the incidence of transection of the RotaWire is not derived from multicenter registries, but may be approximately 0.4% to 1% [208, 209]. There are two types of the transection of the RotaWire: One is the transection at the radiopaque part of the RotaWire, and the other is the transection at the radiolucent part of the RotaWire. The transection of the radiopaque part is easy to notice. If there were the transection of the radiopaque part of the RotaWire, operators should exchange the broken RotaWire to the new one. Since the retrieval of a transected radiopaque fragment of the RotaWire would be similar to that of the conventional guidewire, operators might try retrieval procedures such as twin guidewire method [208]. If the transected fragment of the RotaWire located at the far distal segment of the treated vessel, operators might leave it at the distal segment rather than retrieval. On the other hand, the transection of the radiolucent part of the RotaWire is very difficult to notice. If operators could not notice the transection of the radiolucent part of the RotaWire, operators would have a vessel perforation [209–211]. In fact, Wang et al. reported that the Rotawire damage with subsequent transection was the cause in 18.2% of cases with coronary perforations [182]. Moreover, there would be a greater risk in retrieval of a transected fragment of the RotaWire, if the transection occurred at the radiolucent part. Because the proximal part of the transected fragment of the RotaWire would be sharp, the invisible sharp fragment of the RotaWire may damage to the proximal vessel wall, even to the aortic wall during the retrieval. IVUS may be helpful to identify the invisible fragment. Therefore, it may be safer to seal the transected RotaWire by stenting rather than to try retrieval procedures, especially when the transection occurred at the radiolucent part.

Since the incidence of transection of the RotaWire is low, the causes of transection have not fully understood. Because the metallic fatigue would be the possible cause of transection, it may be important to avoid the continuous contact between the burr and the specific part of the RotaWire. If the RotaWire kinked at the outside of the guide catheter, the transection of RotaWire would happen when the burr advanced over the kinked RotaWire [132]. Furthermore, the WireClip Torquer (Boston Scientific, Marlborough, MA, USA) should be equipped with the RotaWire during the dynaglide mode as well as the high-speed mode to avoid the spinning of the RotaWire, which could be a cause of transection of the RotaWire.

## Complications: Disconnection of the burr and drive shaft fracture

Disconnection of the burr is very rare complications, and there has been no literatures mentioning the incidence of disconnection of the burr. The exact reasons or mechanisms of disconnection of the burr have not been specified. Even senior RA operators with abundant experiences may not or may have a few cases with this complication. Theoretically, the disconnection of the burr could happen when operators activate the burr after the burr was entrapped, or when operators advanced the burr despite strong resistance within the stented segment or angulated segment. Operators may notice the disconnection of the burr by the loss of coordination between the burr motion and the nob motion. The percutaneous bailout may be possible if the RotaWire is not transected, because the 0.014-inch spring tip of the RotaWire may work as the anchor [212, 213]. Imamura S, et al. reported a case of the disconnection of the burr, which was successfully treated percutaneously [214]. In their case report, since the simple pull-back using a guide extension catheter did not work, they sandwiched the disconnected burr between the inflated balloon and the guide extension catheter, and then pulled back together [214]. However, surgical bailout should be considered to this rare complication [215].

Drive shaft fracture is also a very rare complication during RA [216, 217]. Although the exact mechanisms of drive shaft fracture have not been specified, severe tortuosity as well as severe calcification might be associated with drive shaft fracture. Fatal vessel perforation can happen in the case of drive shaft fracture [217].

**Fig. 3:**
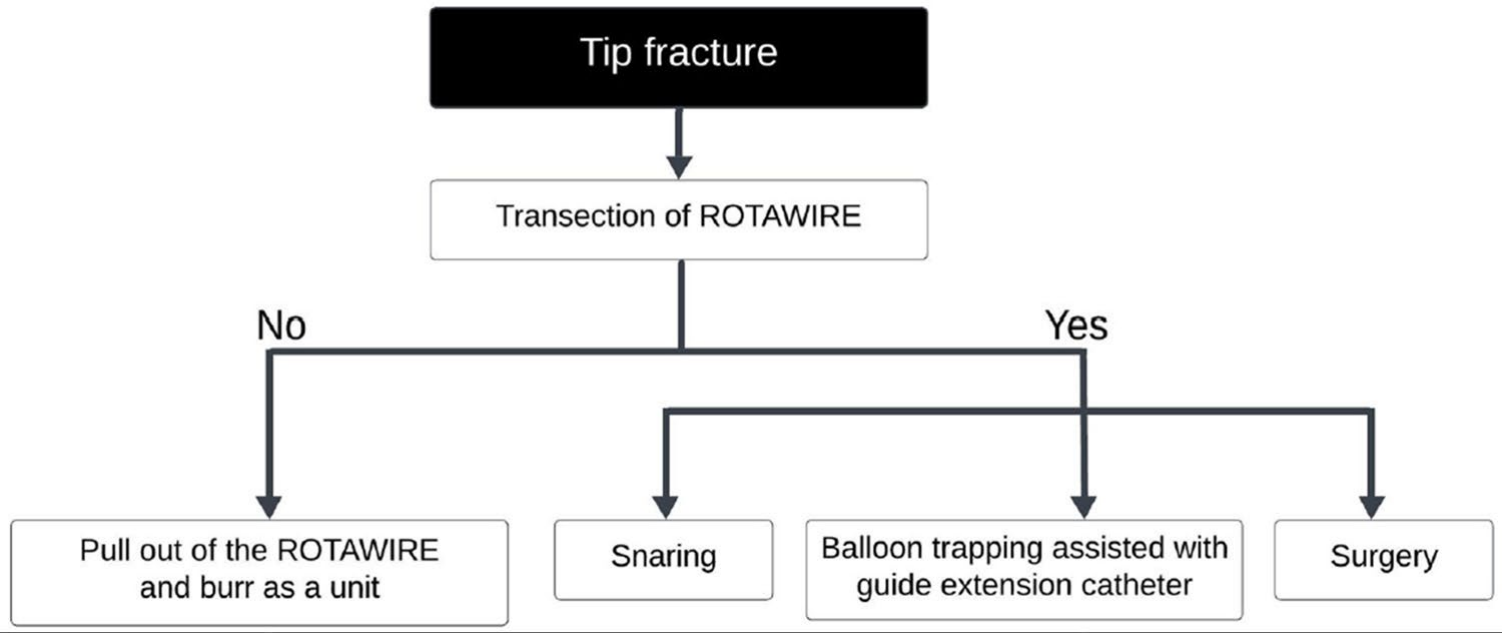
Management strategy for rotablator tip fractures. This figure was reproduced with the permission from Kaneko, et al. [120]

**Fig. 4:**
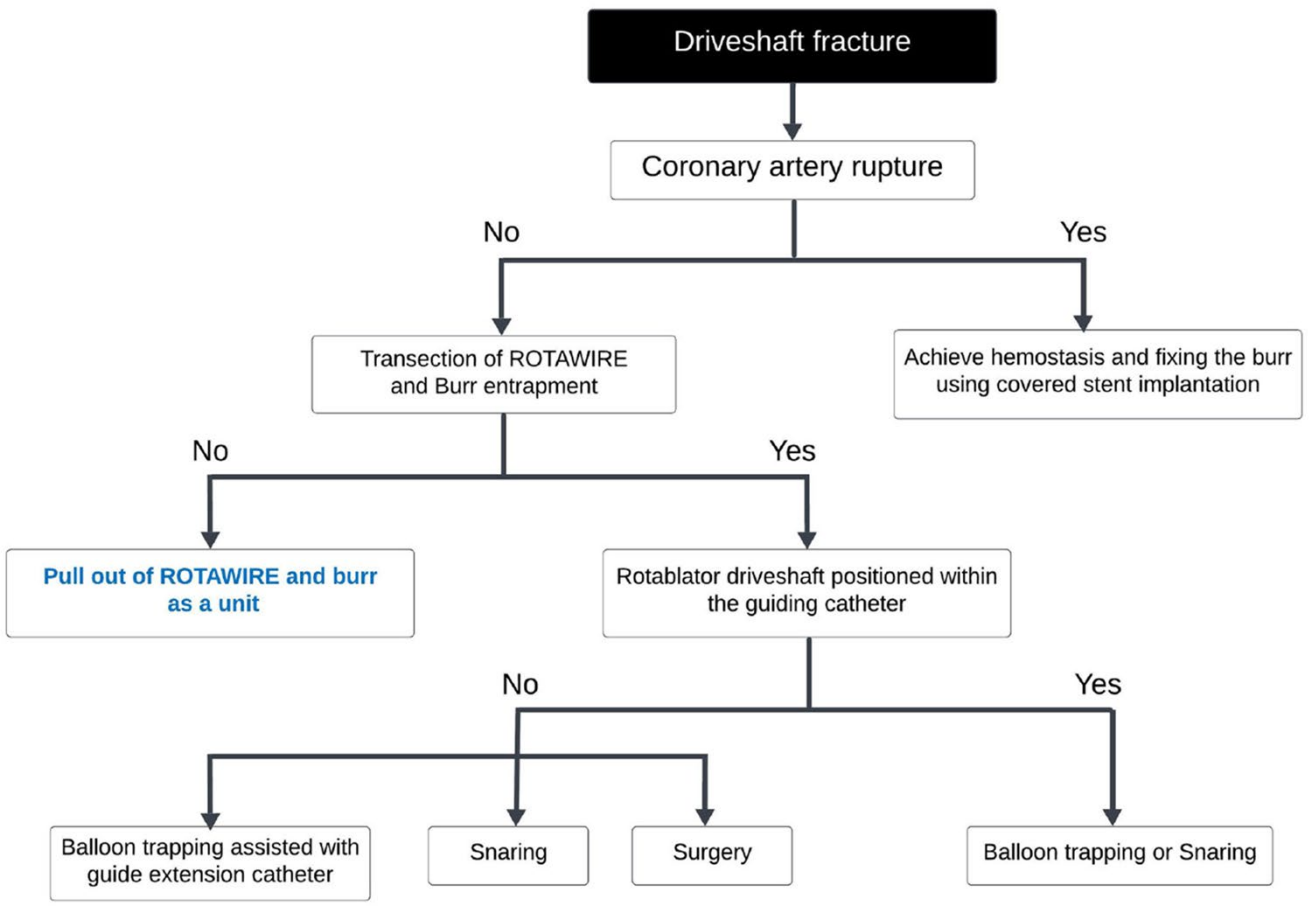
Management strategy for rotablator driveshaft fractures. This figure was reproduced with the permission from Kaneko, et al. [120]

Kaneko, et al. proposed two mechanisms of burr fracture (both disconnection of the burr and drive shaft fracture) without burr entrapment as [1] frequent prolapse during ablation, attributed to significant proximal vessel tortuosity or enlargement, as well as misalignment between the RA device and the target vessel and [2] excessive flexion and loading at the junction between the burr and the driveshaft, or on the driveshaft itself [120]. Also, three pre-fracture signs were proposed as [1] incoherent movement between the advancer’s knob and the burr, [2] visualization of fluoroscopic translucency in the area where the fracture begins to occur (translucent sign), and [3] frequent prolapse of the driveshaft of the RA device in the proximal enlargement or free space [120]. The management algorisms for disconnection of the burr (tip fracture) and drive shaft fracture are shown in Figs. 3 and 4, respectively [120]. Yamaji, et al. also reported a case series of burr disconnection [121], and showed that a gap between the burr and the drive shaft was occasionally observed immediately before burr disconnection, which was termed as the “blank sign” [121]. Both groups (Kaneko, et al. and Yamaji, et al.) emphasize that it is important for the prevention of burr disconnection to recognize the sign of pre-disconnection [120, 121].

Conservative options (leave the burr with or without stenting) may be available if all conditions are satisfied (no luminal obstruction, no risk for migration/embolization of device, no drive shaft/RotaWire extended into aorta, and no coronary ischemia) [197, 218].

## Specific lesions: RCA ostial lesions

Since clinical outcomes of RCA ostial lesions have been unsatisfactory for decades [219], RA has been considered to be a good indication for RCA ostial lesions with severe calcification [83]. However, a good indication does not necessarily mean an appropriate lesion for junior RA operators. Sakakura, et al. reported that the excessive speed reduction during RA was significantly associated with RCA ostial lesions [84], which suggests the difficulty of RA for RCA ostial lesions. There are several reasons why RA for RCA ostial lesions are difficult. First, it is impossible to insert the guide catheter to RCA coaxially. Operators would try to keep guide catheter coaxial to RCA in aorta. Second, coaxiality cannot be confirmed by LAO view, which is a standard view in RA for RCA ostial lesions. Coaxiality is usually confirmed by RAO view in conventional PCI. If the guide catheter can engage to the RCA, coaxiality would be kept once operator check in the RAO view. However, if the guide catheter cannot engage to the RCA, coaxiality would not be kept during procedures. Because it is difficult to check the burr motion in RAO view due to the angiographical shortening of the RCA ostial lesion, the frequent switch between RAO view and LAO view may be necessary to keep coaxiality during RA. Another option is to use straight cranial view, which allows operators to check coaxiality without angiographical shortening of the RCA ostial lesion.

**Fig. 5:**
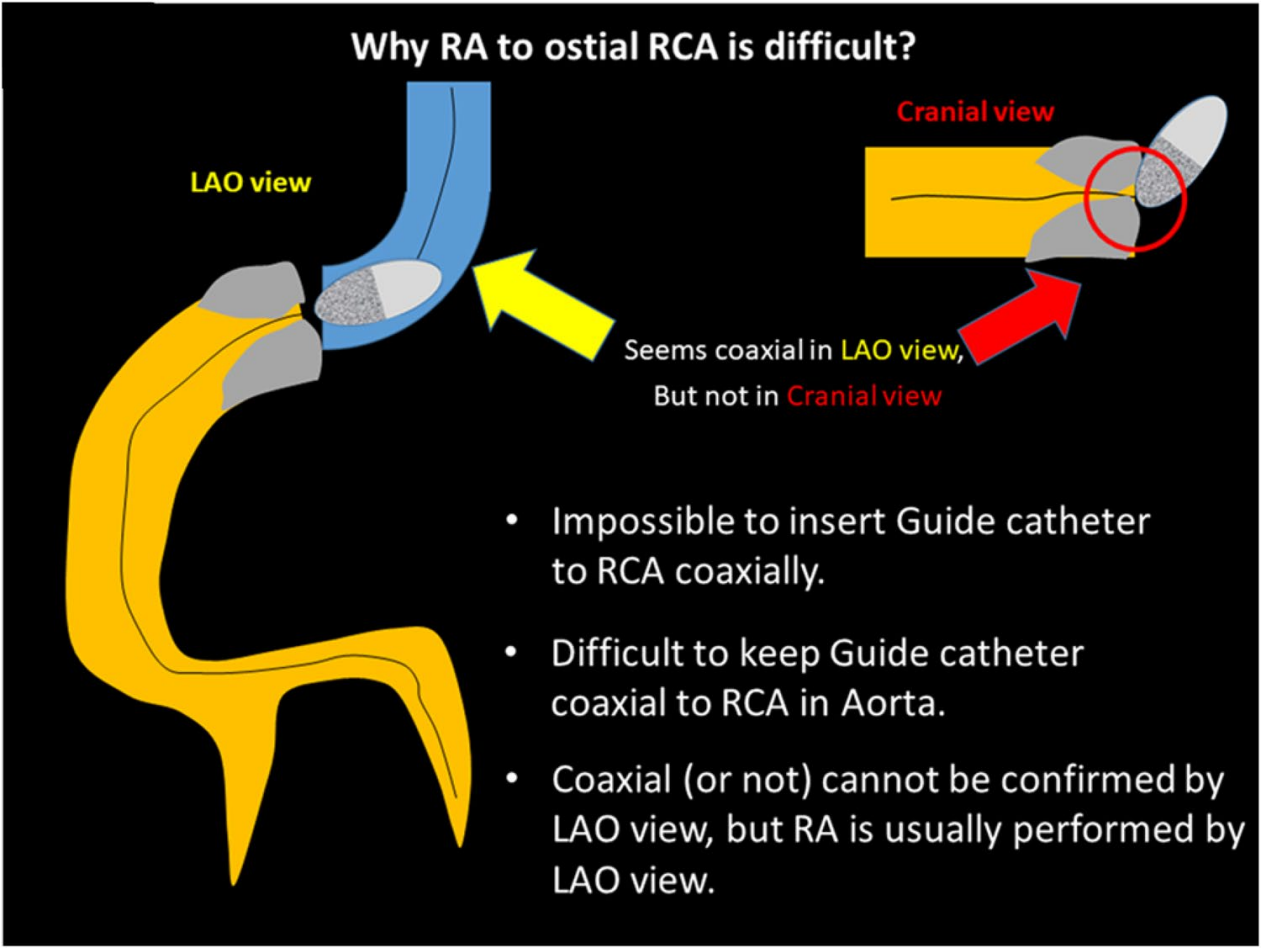
Why RA to ostial RCA is difficult?

The selection of the RotaWire for RCA ostial lesions is also important. The RotaWire Extra-Support may be preferable when operators intentionally remove the guide catheter from the RCA ostium, whereas the RotaWire Extra-Support may be dangerous when operators cannot take a coaxial position. IVUS may help to estimate the risk of RA, especially when operators could not take a coaxial position. Although an IVUS catheter may not cross the lesion before RA, an IVUS catheter would cross the lesion after the crossing of small burrs. IVUS should be tried before using the big burrs for RCA ostial lesions. Furthermore, there may be an additional risk of cerebral infarction following RA for RCA ostial lesions. Guide tip damage may be observed following RA for RCA ostial lesions [220]. It may be important for the prevention of cerebral infarction to use small burrs for an initial attempt in order to minimize the size of debris. Figure 5. summarized the why RA to ostial RCA is difficult. RA for RCA ostial lesions is not recommended for junior RA operators without senior RA operator’s back-up.

## Specific lesions: LCX ostial lesions with substantial bending

**Fig. 6:**
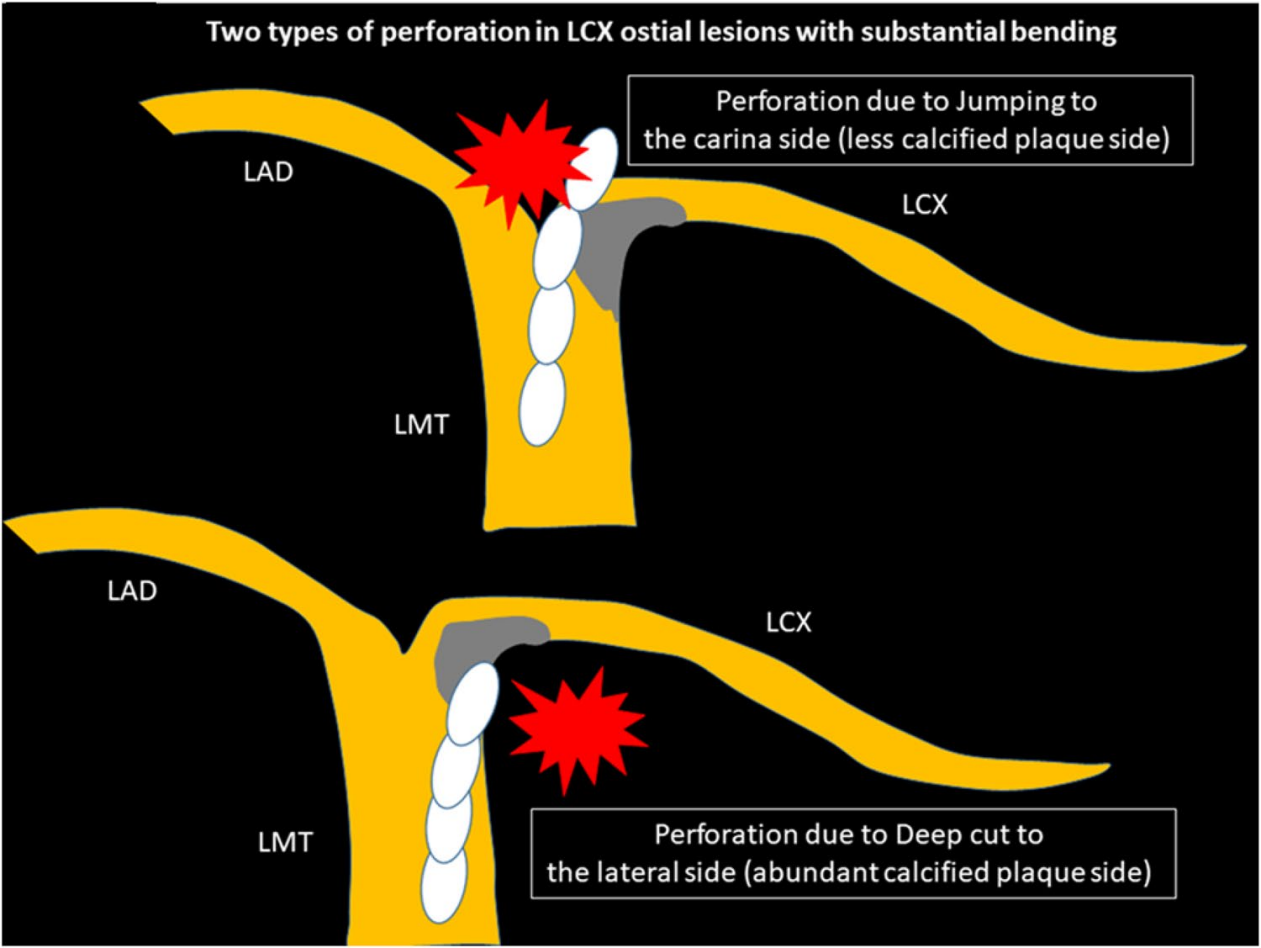
Schema of two types of perforation in LCX ostial lesions with substantial bending

Severely calcified left circumflex (LCX) ostial lesions with substantial bending may be the highest risk lesion in RA. The key to success would be the interpretation of pre-procedural intravascular imaging. If the pre-procedural imaging device could cross the lesion and provide sufficient information including the guidewire bias, operators could select appropriate RotaWires and burrs. Since the tip of guide catheter is close to the LCX ostial lesions, the selection of appropriate guide catheters is also important [221]. Balloon dilatation with small balloons (1.5 mm) may be useful to achieve pre-procedural imaging or to shorten the total ablation time without increasing the risk of severe coronary dissection [221]. However, if the pre-procedural imaging device could not cross, operators need to select RotaWires and burrs from the only angiographical information. In general, atherosclerotic plaques are observed in the lateral wall, whereas atherosclerotic plaques are spared in the flow divider regions (carina) [222]. If severe eccentric calcification was observed in the lateral wall of the LCX ostium, there would be a risk of perforation in the carina side of the LCX ostium due to the jumping of the burr. Operators would try to ablate the lateral wall of the LCX ostium to avoid perforation in the carina side. However, if operators ablated the lateral wall too much, there would another risk of perforation in the lateral wall side due to the deep cut. Two types of perforation in LCX ostial lesions with substantial bending are illustrated in Fig. 6. Operators need to select appropriate burr size, RotaWires, and burr motion to prevent the above two types of perforation. In angiography, it would be important to evaluate the actual contact point between the calcification and the RotaWire using multiple projections. Although low-dose radiation angiography is important for patient’s safety, excessive low-dose radiation angiography may sacrifice the visibility of RotaWires or calcification. Since the visibility of RotaWires or calcification is the cornerstone for high-risk RA, operators should set the radiation dose not to prevent the visibility of RotaWires or calcification. Moreover, if there would be a perforation in the LCX ostium, the percutaneous bailout would be difficult, because the implantation of covered stents may occlude the left anterior descending artery (LAD). Therefore, the indication as well as the strategy of RA to the LCX ostium should be carefully discussed. RA for LCX ostial lesions with substantial bending is not recommended for junior RA operators without senior RA operator’s back-up.

## Specific lesions: unprotected left main lesions

RA to the unprotected left main lesions requires special attention, because slow flow may cause hemodynamic collapse. However, Fuku et al. compared clinical outcomes of left main PCI between with RA (*n* = 108) and without RA (*n* = 1091), and showed the similar rate of complications in left main PCI between with RA and without RA [223], suggesting the acceptable safety in PCI to left main lesions using RA. However, Bouisset, et al. revealed the higher in-hospital mortality in patients who underwent RA to left main in comparison of that in patients who underwent RA to non-left main [224]. In clinical practice, it would be important to start with small burrs (≤ 1.5-mm burr) to prevent fatal slow flow. However, the ≤ 1.5-mm burr may be too small as the final burr size considering the vessel diameter of left main lesions. It would be important to use IVUS/OCT to select an initial burr size and final burr size. If the severe calcified plaques exist at the ostium of left main, the procedure would be more complex as compared to calcified plaques at the middle of left main. In that situation, the stabilization of guide catheter at co-axial position within aorta would be the key to success like the ostium of RCA lesions. Furthermore, the use of IABP and/or temporary pacing should be considered. RA for left main lesions is not recommended for junior RA operators without senior RA operator’s back-up.

If we do not limit the topic to the left main lesions, the subgroup analysis of the PREPARE-CALC study revealed that the incidence of side branch compromise was less frequently observed in lesions treated with RA than in lesions treated without RA in bifurcation lesions [225]. Mizuno, et al. also reported that the incidence of side branch compromise was less frequently observed in calcified bifurcation lesions with RA than in those without RA, even if RA was performed to the main vessel only [226]. On the other hand, Chen, et al. reported the higher risk of side branch perforation, when RA was performed to both main vessel and side branch as compared to RA to the main vessel only [227]. Therefore, RA to both main vessel and side branch should be reserved for senior RA operators.

## Specific lesions: stent ablation

Stent ablation is applied to the restenosis lesions caused by under-expanded stents. Stent underexpansion can occur even after the use of atherectomy devices [228]. Okamura, et al. described a case of stent ablation to treat restenosis lesions due to crushing of a sirolimus-eluting stent [229]. In their report, they performed an experimental study and found that the size of the metallic particles generated during RA of stent struts was 5.6 ± 3.6 μm, which suggested the safe size in a human body [229]. Tips for stent ablation are [1] to select the appropriate burr size, [2] to ablate only stent struts first, [3] and then ablate the calcification behind stent struts using the second burr (size up). Since the risk of burr entrapment is greater during stent ablation [213, 230], gentle manipulation with gradual size-up would be important. Furthermore, stent ablation might be associated with the dysfunction of coronary microcirculation [231]. Although stent ablation might be a single solution for the restenosis lesions caused by under-expanded stents, the long-term outcomes were not satisfactory [232–235]. Coronary lithotripsy may be another option for stent underexpansion due to severe calcification [236–238], while this treatment is off-label and sufficient follow-up data is not available yet. Stent ablation is not recommended for junior RA operators. Furthermore, on-site surgical back-up may be needed because of greater risk of burr entrapment, when operators perform stent ablation using big burrs. Since in-stent calcification with or without stent underexpansion is closely associated with poor clinical outcomes and is difficult to treat even by RA [239–241], there is an emerging idea that drug-coated balloon keeps more options at the treatment of restenosis than stenting in the treatment of severely calcified lesions [51, 242–245].

## Specific lesions: diffuse long lesions

**Fig. 7:**
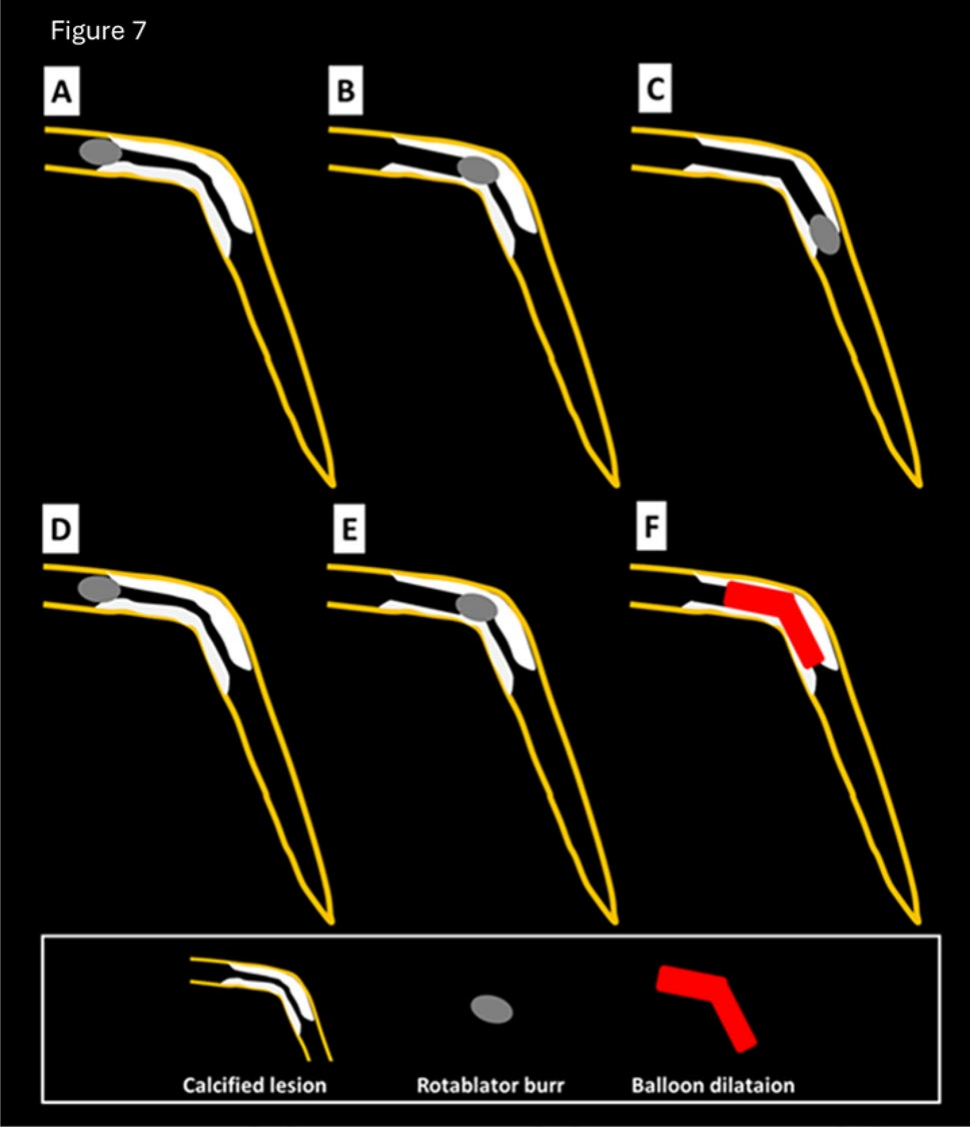
Schema of conventional and halfway rotational atherectomy Panels **(A)**, **(B)**, and **(C)** illustrate the conventional rotational atherectomy, whereas panels **(D)**, **(E)**, and **(F)** illustrate the halfway rotational atherectomy. **(A)**: The burr positioned just before the calcified lesion. **(B)**: The burr ablated the proximal segment of the calcified lesion. **(C)**: The burr ablated the full segment of the calcified lesion. **(D)**: The burr positioned just before the calcified lesion. **(E)**: The burr ablated the proximal segment of the calcified lesion. **(F)**: Balloon dilatation was performed for the rest of the calcified lesion. This figure was reproduced with the permission from Sakakura, et al. [168]

RA was frequently performed for diffuse long lesions, and the long-term outcomes of long lesions treated by RA were comparable to those of short lesions treated by RA [246]. However, RA for diffuse long lesions may be difficult for junior RA operators, especially when there is an angle in the middle of diffuse long lesions. The presence of an acute angulation on the lesion is reported to be associated with procedural failure of RA [247]. Sakakura, et al. reported the utility of halfway RA especially for lesions with an angle [168, 248]. In brief, the operator does not advance the burr beyond the angle within the lesion to avoid burr entrapment or vessel perforation, and balloon dilatation is performed beyond the angle after RA (Fig. 7). Halfway RA may be useful especially for junior RA operators. If halfway RA did not work (e.g. balloon could not dilate the distal calcified lesion), switch from halfway RA to conventional RA should be considered. Although the other option could be to use the guide extension catheter, RA beyond the guide extension catheter is not straightforward. Another important aspect in RA for diffuse long lesions is to check the speed down of rotational speed. Although the excessive speed down during RA should be avoided, absence of reasonable speed down may mean that the burr does not contact calcification adequately. If an operator pushes the burr too much in the absence of speed down, there would be substantial risk of burr entrapment or vessel perforation. Therefore, an operator may change the RotaWires to facilitate the contact between the burr and the calcified plaques, switch to balloon dilatation (resultantly halfway RA), or rarely burr size-up to increase the contact area in the absence of reasonable speed down [184, 249].

**Fig. 8:**
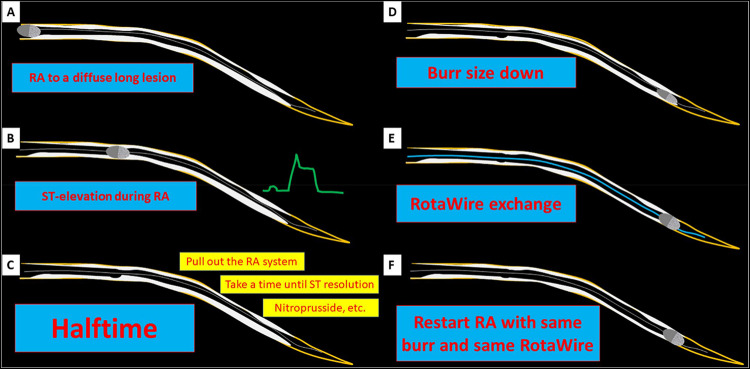
Representative illustration of halftime rotational atherectomy. Panel **A**. Rotational atherectomy (RA) to a diffuse long lesion is performed. Panel **B**. A marked ST-segment elevation is observed during RA. The burr is in the middle of the lesion. Panel **C**. The RA system including the burr is pulled out from the guide catheter. An operator takes a halftime until ST resolution. Intracoronary vasodilators such as nitroprusside can be injected. If coronary flow is fully recovered, an operator can select different approaches (Panels **D**, **E**, F). Panel (D) The burr-size down is a standard approach for diffuse long calcified lesion. Panel (E) The exchange of RotaWire is also a standard approach for diffuse long calcified lesion. Panel (F) The restarting RA with same burr and same RotaWire is a unique approach for diffuse long calcified lesion. This approach does not require additional cost. This figure was reproduced with the permission from Sakakura, et al. [169]

**Fig. 9:**
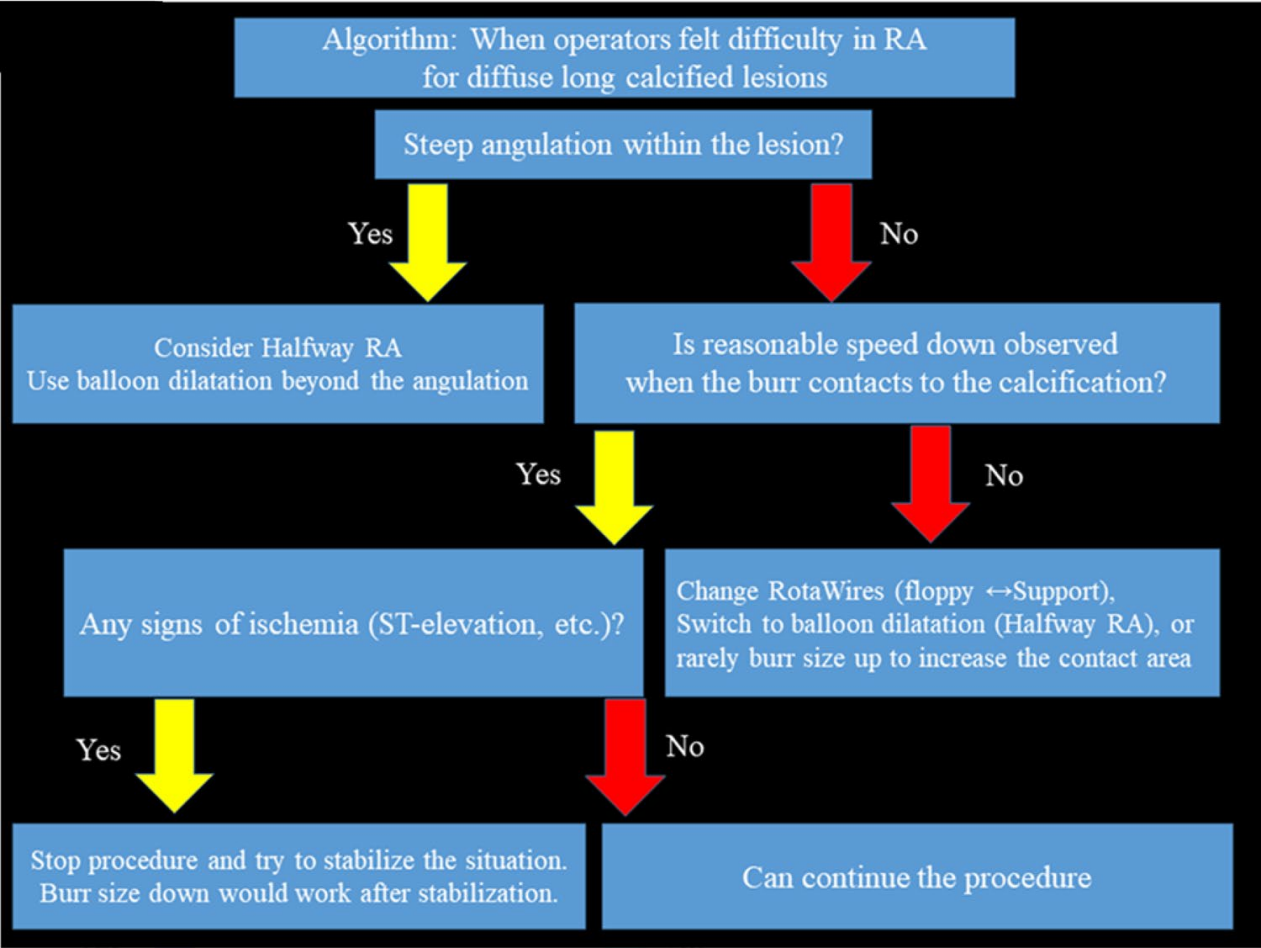
Algorithm: When operators felt difficulty in RA for diffuse long calcified lesions

Operators should also take care of ischemia during RA to the diffuse long lesions, because the risk of slow flow is greater in the diffuse long lesions than the short lesions [124]. If operators noticed the signs of ischemia in the middle of diffuse long lesions, size down of the burr would be the reasonable choice. Another option would be to take a short break with removing the burr from the coronary artery in order to stabilize coronary flow and vital signs. If coronary flow is restored and vital signs are stabilized, operators can safely resume procedures. Sakakura, et al. proposed the utility of halftime RA when ST-segment elevation is observed during RA to the diffuse long lesion [169]. Although halftime RA includes burr-size down, exchange of RotaWire, and both (size-down and exchange of RotaWire), the main concept of halftime RA is [1] to pull out the RA system and take a sufficient time until ST-segment resolution, [2] to inject intracoronary vasodilator directly and perform intravascular imaging during halftime, and [3] to restart RA with same burr size and same RotaWire when the complete ST-segment resolution is observed [169] (Fig. 8). This document suggests an algorithm for diffuse long lesions, especially for junior RA operators (Fig. 9).

The maximum working range of the burr is approximately 70 mm. Operators usually set the nob at 1–2 cm apart from the end, and set the platform at 1–2 cm proximal from the lesion. Thus, the actual working range of the burr is approximately 30–50 mm. If the length of the target lesion was beyond 30–50 mm, operators need to move the platform during the procedure. Although there were few literatures regarding how to move the platform during the procedure, there were several ways among RA experts. The most important point is to ablate the proximal part of the diffuse long lesion sufficiently before moving the platform. Only 1 pass would not be enough. Several passes would be necessary until no additional speed down was observed. Some experts prefer to size up the burr and ablate the proximal part of the diffuse long lesion using big burrs to make a stable platform. After operators ablated the proximal part of the diffuse long lesion sufficiently, operators could move the platform more distally. Some experts prefer to use dynaglide mode to move the platform. Sliding sheath technique, in which operators park the burr to the distal end and then bring the advancer to follow the burr, may be used by some experts. Since the outer diameter of the drive shaft sheath is 4.3 Fr (1.43 mm), the ablation by the burr-1.25 mm may not be enough to move the platform.

It is sometimes difficult to deliver the burr into the distal target segment beyond the proximal tortuosity. In those situations, the use of guide extension catheters is an option. However, the advancement of RA burr through the guide extension catheter may fail at the entry port of the guide extension catheter. Lumen collapse in tortuous or calcified vessels may limit burr delivery [250]. Kaneko, et al. proposed the DELIVER (Deep Engagement of guide catheter or 5-F chiLd-guIde catheter for burr deliVEry and subsequent Rotational atherectomy) technique, which ensures smooth delivery of the RA burr within the catheter, in contrast to the challenges experienced with the guide extension catheter [251, 252].

## Specific lesions: ablation to an entrapped guidewire

An entrapment of coronary guidewires is sometimes observed in complex PCI. Forced pull-back of the entrapped guidewire may damage coronary artery wall or provoke the fracture of guidewire. RA can be an option to cut the entrapped guidewires [253–256]. In the comparison of forced pull-back by snare, the coronary artery is not subjected to excessive force if the contact point between the burr and the fractured guidewire is controlled under IVUS guidance [254]. However, since there is a potential risk that the RA burr may be entrapped in the spring wire, ablation to an entrapped guidewire is not recommended for junior RA operators.

## Conclusions

In this document, we provided the Japanese style RA, which uses intravascular imaging devices to achieve the maximum efficacy without sacrificing the safety. Our recommendation focused on mainly junior RA operators but would be helpful for senior RA operators for their more advanced procedures.
